# The application of Vavilov’s approaches to the phylogeny and evolution of cultivated species of the genus Avena L.

**DOI:** 10.18699/VJGB-23-107

**Published:** 2023-12

**Authors:** I.G. Loskutov, A.A. Gnutikov, E.V. Blinova, A.V. Rodionov

**Affiliations:** Federal Research Center the N.I. Vavilov All-Russian Institute of Plant Genetic Resources (VIR), St. Petersburg, Russia Saint Petersburg State University, St. Petersburg, Russia; Federal Research Center the N.I. Vavilov All-Russian Institute of Plant Genetic Resources (VIR), St. Petersburg, Russia; Federal Research Center the N.I. Vavilov All-Russian Institute of Plant Genetic Resources (VIR), St. Petersburg, Russia; Saint Petersburg State University, St. Petersburg, Russia Komarov Botanical Institute of the Russian Academy of Sciences, St. Petersburg, Russia

**Keywords:** Avena species, сenter of origin, itraspecific diversity, law of homologous series, NGS methods sequences, VIR global collection, виды овса, центры происхождения, внутривидовое разнообразие, закон гомологических рядов, NGS секвенирование, мировая коллекция ВИР

## Abstract

The central problem that Vavilov was investigating was the overall concept of global plant genetic resources. The theoretical basis of this concept consisted of the law of homologous series in variation, research on the problem of species as a system, botanical and geographical bases of plant breeding, and the key theory of the centers of origin of cultivated plants. The VIR global collection of plant genetic resources collected by Vavilov and his associates from all over the world reflects the fullness of botanical, morphological and genetic diversity, and can be used for historical, evolutionary, phylogenetic and applied breeding research aimed at unlocking the potential of all the collection material. The whole diversity of cultivated oats, as was proved by Vavilov, had originated from segetal weeds. This process can be clearly traced in Spain on the example of the cultivated diploid species A. strigosa, A. abyssinica in Ethiopia, A. byzantina in Turkey and Iran, and on segetal forms of A. sativa. The studies of the morphological features as a whole do not yield a complete picture of the evolutionary and systematic status of some oat species and forms. The methods and approaches that use DNA markers and genomic technologies, and are promising for the study of oat polymorphism and phylogeny have been actively researched recently. A number of works devoted to the molecular aspects of the evolution and phylogeny of the genus Avena have recently appeared. The research uses various markers of genes, gene regions, intergenic spacers (internal and external), both nuclear and chloroplast and mitochondrial, genomic approaches and other modern methods. On the basis of a comprehensive study of the complete intraspecific diversity from different zones of the distribution range of cultivated oat species as well as on the basis of an analysis of data on the geography of forms and species distribution ranges, it was established that the process of hexaploid species formation also took place in the western part of the Mediterranean, and subsequently, when moving eastward, these forms started occupying all the vast spaces in the region of the Southwest Asian center, forming a large intraspecific diversity of wild forms and weedy ones in transit to cultivated hexaploid oat species. An analysis of the intraspecific diversity of landraces has specified the centers of morphogenesis of all cultivated oat species. The phylogenetic analysis of the representative intraspecific diversity of cultivated and wild Avena species carried out using next generation sequencing (NGS) showed that diploid species with A-genome variants are in fact not primary diploids, but a peculiar Mediterranean introgressive hybridization complex of species that sporadically enter into interspecific hybridization. It was established that the tetraploid cultivated species A. abyssinica had most likely originated from the wild A. vaviloviana. An analysis of the ways of A. sativa and A. byzantina domestication showed that the most widespread ribotype of the A. sativa hexaploid was inherited from A. ludoviciana, and the second most widespread one, from A. magna, while A. byzantina has two unique ribotype families, most likely inherited from an extinct oat species or a still undiscovered cryptospecies.

## Abstract (ru)

Центральной проблемой, которую исследовал Н.И. Вавилов, было учение о мировом генофонде куль- турных растений. Теоретическую основу этого учения составили: закон гомологических рядов в наследственной изменчивости, разработка проблемы вида как системы, ботанико-географические основы селекции и теория цен- тров происхождения культурных растений. Собранная Н.И. Вавиловым и его соратниками со всех уголков мира коллекция генетических ресурсов растений ВИР, представляющая всю полноту ботанического, морфологического и генетического разнообразия, позволяет проводить исторические, эволюционные, филогенетические и прикладные селекционные исследования, направленные на раскрытие потенциала всего коллекционного материала. Положе- ния Н.И. Вавилова по комплексному анализу всего видового и внутривидового разнообразия культурных и диких видов дают возможность сделать верные выводы при изучении сложных экологически дифференцированных ви- довых систем, связанных в своем формировании с определенной средой и воздействием отбора. Все разнообразие видов культурного овса, как было доказано Н.И. Вавиловым, имеет сорно-полевое происхождение. Этот процесс можно наглядно проследить в Испании – на примере культурного диплоидного вида Avena strigosa, в Эфиопии – A. abyssinica, в Турции и Иране – A. byzantina и на сорно-полевых формах A. sativa. Изучение комплекса морфологиче- ских признаков не дает полного представления об эволюционном и систематическом положении некоторых видов и форм овса. Для исследования полиморфизма, филогении и эволюции овса перспективны активно разрабатываемые в настоящее время методы и подходы с использованием ДНК-маркеров и геномных технологий. Появился ряд работ, затрагивающих молекулярные аспекты эволюции и филогении рода Avena. В исследованиях используют раз- личные маркеры генов, участков генов, межгенных спейсеров (внутренних и внешних), как ядерных, так и хлоро- пластных и митохондриальных, геномные подходы и другие современные методы. На основе комплексного изучения полного внутривидового разнообразия из разных зон ареала культурных видов овса и анализа данных по географическому распределению ареалов форм и видов установлено, что процесс формирования гексаплоидных видов шел также в западной части Средиземноморья, и затем при продвижении на восток эти формы стали за- нимать значительные пространства в районе Юго-Западного Азиатского центра, образуя большое внутривидовое разнообразие диких и переходных сорных форм к культурным видам гексаплоидного овса. В результате анализа внутривидового разнообразия староместных сортов были уточнены центры формообразования всех культурных видов овса. Осуществленный с помощью метода секвенирования следующего поколения (NGS) филогенетический анализ представительного внутривидового разнообразия культурных и диких видов рода Avena показал, что ди- плоидные виды с вариантами генома А в действительности являются не первичными диплоидами, а своеобразным средиземноморским интрогрессивно-гибридизационным комплексом видов, спорадически вступающих в межви- довые скрещивания. Установлено, что тетраплоидный культурный вид A. abyssinica, вероятнее всего, происходит от дикого вида A. vaviloviana. Анализ путей одомашнивания культурных видов овса A. sativa и A. byzantina показал, что наиболее массовый риботип гексаплоида A. sativa унаследован от A. ludoviciana, а второй по массовости – от A. magna, в то же время A. byzantina обладает двумя уникальными семействами риботипов, скорее всего, унаследо- ванными от вымершего вида овса или криптовида, до сегодняшнего дня не обнаруженного.

## Introduction

When considering the scientific heritage of Nikolai Ivanovich Vavilov, it is notable how his studies as a plant grower, breeder, botanist and ethnographer are intertwined and complement each other. It is impossible to draw boundaries between his works on breeding, plant growing and genetics. This feature of his scientific style is of great importance, as it marks a turn in theory and research methods. He always took new paths and regarded the world of plants he was studying from a new, still unknown point of view.

The central problem N.I. Vavilov was investigating was the overall concept of the global diversity of plant genetic resources. It included a number of his major theoretical generalizations, which determined new paths in the theory of introduction and applied botany, brought world fame to Vavilov and played a prominent role in the development of genetics and agricultural crop breeding throughout the world. The theoretical basis of this concept was the law of homologous series in variation, developments of the problem of species as a system, botanical and geographical foundations of breeding, and the theory of the centers of origin of cultivated plants (Loskutov, 1999, 2009).

The main ideas that were dominant in N.I. Vavilov’s works were the idea of plant world evolution, and the idea of botanical geography and the sequence of variability stages in space and time, characteristic of cultivated and wild plant species (Vavilov, 1997).

The sources of N.I. Vavilov’s special approach to the study of vast plant material are found in the creative work of his great predecessors, namely Alphonse De Candolle and Charles Darwin. It is noteworthy that Vavilov’s book “Studies on the Origin of Cultivated Plants” (1926) began with the words “Dedicated to the memory of Alphonse de Candolle, author of “Géographie botanique raisonnée”, 1855, “La phytographie ou l’art de décrire les végétaux considérés sous différents points de vue”, 1880, “Origine des plantes cultivées”, 1882”.

In his article “The theory of the origin of cultivated plants after Darwin” (1940), N.I. Vavilov noted that in his approach to the variability and evolution of cultivated plants, Darwin relied primarily on the works of A. De Candolle, but unlike him, Darwin was interested in the evolution of species, in hereditary changes that a species introduced into cultivation had undergone, while De Candolle was interested in establishing the homeland of cultivated plants. Unlike De Candolle, Vavilov, like Darwin, paid great attention to both the main areas of the species origin and the evolutionary stages the species were passing during their spreading influenced by cultivation, environmental conditions, and natural and artificial selection. Based on the main provisions of the theories of Darwin and De Candolle, N.I. Vavilov formulated tasks for research designed for a long period of time. N.I. Vavilov conceived a systematic study of the genetic diversity and origin of the most important crops, encompassing all the evolutionary stages, from the primary areas where connections with wild forms can still be traced and where phylogenetic relationships between various wild species and cultivated forms can be established, tracing further historical distribution of species, up to the final aspects of modern breeding (Vavilov, 1992).

Nikolai I. Vavilov noted that evolution proceeded in space and time; which means that only by closely approaching the geographical centers of morphogenesis, having discovered all the links connecting the species, one can search for ways to master the synthesis of Linnaean species, with the understanding of the latter as systems of forms that have a huge intraspecific diversity of alleles. The problem of speciation itself was considered by Vavilov not as a problem of the formation of separate races, which, according to Darwin, were separating into specific species, but as a process of the origin of complex, genetically and phenotypically diverse populations, representing true Linnaean species, for each of which and for each related group of which its own spectrum of morphological and physiological variability is characteristic (Vavilov, 1992).

The discovery of the centers of origin of cultivated plants by Vavilov in 1926 is so significant, as it opens a possibility of finding in these areas valuable genetic diversity of plant forms that are most adaptive to various environmental conditions and are represented by heterogeneous populations (Vavilov, 1992).

In the primary centers, diverse and sometimes opposite genetic processes can take place simultaneously and independently of each other, leading to a mismatch between the centers of plant origin and the centers of the greatest intraspecific genetic diversity. These are the centers where the majority of dominant alleles of genes are concentrated. The zones of recessive forms concentration are the areas of intense mutational morphogenesis, which are located on the periphery of the centers of origin. An analysis of dominant and recessive forms ratio within species in a certain geographical area can reveal the level of morphogenesis, the rate and stage of species evolution (Vavilov, 1992).

Summing up his fruitful work on speciation, N.I. Vavilov published the work entitled “The Linnean species as a system”, the main provisions of which were reported in 1930 at the 5th International Botanical Congress in Cambridge (Great Britain) (Vavilov, 1931). Here, the concept of the Linnean species as a regular system of forms, phenotypically, physiologically and genetically variable within certain limits, is very significant both for the practical purposes of studying cultivated plants and for studying the main issues of the evolutionary process. It was possible to come close to studying this process only with the understanding of the Linnaean species in its diversity, and not as a monotypic species described from a few specimens, in the way it was customary to describe species. The main problems of evolution could not be resolved without taking into account the species as a complex system of forms (genotypes). The genetics of individual species gives an idea of the hereditary nature of a species only when it is based not on a few random specimens or crop varieties, but on the definitely and carefully chosen, though it may be selective, material (Vavilov, 1992).

The global collection of plant genetic resources collected by N.I. Vavilov and his associates from all over the world reflects the fullness of botanical, morphological and genetic diversity. It has been preserved by the VIR staff in the most difficult periods of history, and now it makes it possible to conduct historical, evolutionary, phylogenetic and applied breeding research aimed at unlocking the potential of all the material in the collection (Loskutov, 2009).

According to Nikolai I. Vavilov, a species is a complex, mobile, isolated morpho-physiological system associated in its genesis with a certain environment and distribution range, subject to the law of homologous series in variation (Vavilov, 1992). To determine the system of a species, it is necessary to study the complete intraspecific diversity from different parts of the distribution area and establish the range of variability of characters in different environmental conditions. These provisions constitute a theoretical basis that makes it possible to predict the discovery of various plant forms and explains how the system of hereditary forms of a species evolves according to growing conditions. The law of homologous series helps to establish solid foundations for the taxonomy of cultivated plants, gives an idea of the place of each systematic unit in the vast wealth of the plant world. Therefore, a real intraspecific classification should be based on an integrated approach to the concept of the rank of a botanical variety as an objective unit of complex polymorphic species systems. N.I. Vavilov emphasized the complexity of the species system as a whole, consisting of connected and mutually penetrating parts, forms and genotypes, in which he points to the facts associated with the genus Avena L. (Vavilov, 1951).

## N.I. Vavilov about evolution and phylogeny of the genus Avena

In his works, Vavilov paid great attention to the evolution and phylogeny of the entire genus Avena L. In 1927, he definitely spoke of four main genetic groups of cultivated oats related by origin: A. sativa L. – A. fatua L.; A. byzantina K. Koch ‒ A. sterilis L.; A. strigosa Schreb. – A. barbata Pott; and A. abyssinica Hochst. Particularly intricate was the first, extremely polymorphic group of A. sativa, the origin of which is associated with Asia (Vavilov, 1992). This point of view began to dominate in all studies, in contrast to the opinion about the European origin of cultivated oats (Ladizinsky, 1989).

From the genetic point of view, oats (Avena L.) have not been sufficiently studied compared to other cereal crops. A systematic study of varietal diversity and individual species of the genus provides general information on the localization of the centers of their morphogenesis, evolution, and domestication (Loskutov, 2007). Avena species are characterized by great morphological and eco-geographical diversity, and landraces are highly adaptable. Since the early 20th century, the world literature has accumulated a significant amount of data on numerous forms and species of the entire genus, and on the centers of their greatest diversity and origin (Malzew, 1930; Baum, 1977; Vavilov, 1992; Rodionova et al., 1994; Loskutov, Rines, 2011; Ladizinsky, 2012).

Landraces, including Mediterranean ones, collected during the expeditions of N.I. Vavilov and his associates, were researched in the 1930–1950s and have not been studied in detail by now (Mordvinkina, 1960). Nowadays, many problems remain completely unresolved; there is no consensus on the origin, systematic status, relationships and ways of cultivation of oat species. Comparative studies of (landraces and segetal) varieties and wild species of oats from the evolutionary, taxonomic and breeding points of view are caused by the great interest of breeders in their practical use.

The determination of the areas of origin and morphogenesis in oat species employs the differential botanicalgeographical method investigated and widely used by N.I. Vavilov. The essence of this method is in the determination of a wide intraspecific diversity when analyzing the differentiation of some plant species into botanical varieties and genetic groups, in elucidating the nature of the distribution of the hereditary diversity of forms of a given species within the distribution range, with the establishment of geographical centers of accumulation of this diversity and geographical localization of the morphogenetic process (Vavilov, 1992). The analysis of collection accessions showed that all the considered forms of oats belonging to individual species were characterized by morphological features and certain distribution ranges.

According to N.I. Vavilov, it is impossible to reduce the origin of cultivated oat species to a single geographical center. Cultivated oat species (diploid and polyploid) are undoubtedly of polyphyletic origin. Some species, in all likelihood, entered cultivation independently. In any case, it would be erroneous to consider cultivated oats definitely associated only with Europe. The presence of endemic hulled and naked groups of A. sativa in China, wide distribution of wild and weedy A. fatua and A. ludoviciana Durieu in Turkestan, Bukhara, Afghanistan, Persia, the Transcaucasus, and Armenia, the presence of many original groups of cultivated and wild oats there testify to the participation of Asia in the formation of the A. fatua – A. ludoviciana – A. sativa group of hulled and naked forms (Vavilov, 1992

The whole diversity of cultivated oats, as shown by Vavilov (Vavilov, 1992), had originated from segetal weeds. With the spread of the species northward or to the highlands, to more harsh and humid growing conditions, oats eventually replaced the main crops (among which it had originally been only a weed plant), and itself became a proper cultivated plant. This process can be clearly traced in Spain on the example of the cultivated diploid species A. strigosa, on A. abyssinica in Ethiopia, A. byzantina in Turkey and Iran, and on segetal forms of A. sativa convar. asiatica (Vavilov) Rodionova et Soldatov and A. sativa convar. volgensis (Vavilov) Rodionova et Soldatov (Loskutov, 2007).

## Intraspecific diversity of cultivated oats species

According to the classification of N.A. Rodionova et al. (1994), the cultivated diploid A. strigosa is divided into three subspecies, i. e. A. strigosa (Schreb.) subsp. strigosa, A. strigosa subsp. brevis (Roth) Husn., and A. strigosa subsp. nudibrevis (Vavilov) Kobyl. et Rodionova, which are clearly geographically differentiated. Of the 15 identified botanical varieties in the entire A. strigosa species, 8 were found among native specimens from Spain and 11 among specimens from Portugal. In total, 13 botanical varieties are found on the Iberian Peninsula, most of which are endemic to this region. The greater part of diverse forms of this species was distributed in Spain, Portugal, Germany and Great Britain; besides, individual forms originated from a number of other European countries. Thus, the center of origin and diversity of the diploid cultivated species A. strigosa is the Iberian Peninsula, where both its wild relatives and probable progenitors, the diploid species A. hirtula Lag. and A. wiestii Steud., are widespread (Loskutov, Rines, 2011). According to archaeological data collected by A.I. Malzew (1930), A. strigosa was the first oat species that was cultivated in Europe already in the Neolithic era, i. e. about 1500 BC.

The tetraploid cultivated species A. abyssinica that infests barley and wheat fields is currently cultivated to a limited extent, although it has a cultural type of caryopses articulation that prevents their shattering when ripe. In addition to this endemic species, only tetraploid species A. vaviloviana (Malzew) Mordv. and A. barbata of the wild ones grow in Ethiopia. A. abyssinica shares many features with A. vaviloviana and is considered to be its cultural counterpart.

All of the small intraspecific diversity of six forms in the rank of botanical varieties of A. abyssinica is found only in the present-day Ethiopia (Rodionova et al., 1994). According to A.I. Malzew (Malzew, 1930), the Ethiopian center of diversity of tetraploid oat species is a secondary one, and the forms distributed in it had links with the Mediterranean center of origin in the early historical epoch. The secondary status of this center is also proved by the fact that two related species, A. vaviloviana and A. abyssinica, have a purely spring type of growing, which is secondary to the winter type of growing. Apparently, these two species, having found the most favorable climatic and soil conditions in Ethiopia, south of the Mediterranean center, spread there and could not advance further due to more harsh arid climatic conditions in the countries adjacent to Ethiopia (Loskutov, Rines, 2011).

The hexaploid cultivated species A. byzantina, according to N.A. Rodionova et al. (1994), numbers 15 botanical varieties, 9 of which were found among landraces from Algeria, 8 from Morocco and Turkey each, 7 from Greece, 6 from Israel, and 5 from Spain and Italy each; the rest of the countries where this species was distributed had from one to three botanical varieties. It was noted by N.I. Vavilov that the main area of diversity of this species is concentrated on the Mediterranean coast of North Africa (Vavilov, 1992). Therefore, the primary center of morphogenesis in A. byzantina is the territory of Algeria and Morocco, where its greatest local botanical diversity is concentrated, while the presence of a large number of intermediate forms in Turkey indicates that this region is a secondary center of diversity for this species (Loskutov, 2007).

A study of the intraspecific diversity of the collection of the hulled forms of the hexaploid cultivated species A. sativa L. showed that segetal forms of this group of botanical varieties, numbering about 130 landraces in the N.I. Vavilov All-Russian Institute of Plant Genetic Resources (VIR) collection, are localized on the territory of Iran, Georgia and the Russian Federation (Dagestan, Tatarstan, Bashkortostan and Chuvashia). This group of forms weeding crops was characterized by primitive or transitional features and had a clear confinement to certain distribution ranges. An analysis of the data on the composition of botanical variety of landraces in the collection showed that the forms of A. sativa subsp. sativa convar. asiatica (Rodionova et al., 1994) demonstrated the greatest diversity only in Iran and Georgia, where all three botanical varieties characterizing this group were identified, while in the Russian Federation (Dagestan), only one botanical variety from this group was identified.

In addition, a form belonging to a botanical variety from the group A. sativa subsp. sativa convar. volgensis (Rodionova et al., 1994) and representing a link between the two groups of varieties was found here. The group of A. sativa subsp. sativa convar. volgensis itself has four botanical varieties, the greatest diversity of which is confined to the Russian Federation. All four botanical varieties were found in Tatarstan, three were found in Bashkortostan, Chuvashia and Ulyanovsk province, two in Udmurtia, and only one in Kirov and Saratov provinces, and Mordovia each. In other regions of distribution of hulled oats, these forms are not found. Apparently, it was from the South-Western Asiatic center via Iran and further through Georgia into the Russian Federation (Dagestan, Saratov and Ulyanovsk provinces, Tatarstan, Chuvashia, and Bashkortostan) that the hulled forms of A. sativa subsp. sativa started first weeding crops, then entering cultivation and spreading in all directions (Loskutov, 2007).

Another subspecies, A. sativa subsp. nudisativa (Husn.) Rodionova et Soldatov, or naked forms of hexaploid oats (Rodionova et al., 1994), originated from China, as stated by Vavilov (Vavilov, 1992). It is known from the literary sources that naked oats were widespread in China already in the 5th century AD (Zukovskij, 1962). Eastward from the main center of diversity, the growing conditions changed, resulting in the appearance of naked mutations of A. sativa, which settled in new habitats. Thus, a cycle of transition of forms of the wild, cultivated hulled and naked types is observed here for both A. sativa and A. strigosa. The last type, being a recessive mutation, appears at a distance from the territory of the main diversity of the closely related hulled forms.

An analysis of data on intraspecific diversity of landraces of naked cultivated hexaploid species A. sativa, numbering over 40 accessions, showed that out of four botanical varieties identified in the VIR collection (Rodionova et al., 1994), all four were identified among accessions from Mongolia, three among those from China and two among those from the adjacent Krasnoyarsk Territory in the Russian Federation (Loskutov, 1999). Two most common botanical varieties A. sativa subsp. nudisativa var. inermis Koern. and A. sativa subsp. nudisativa var. chinensis Doell. are characteristic of accessions from other regions. Consequently, the center of diversity of naked hexaploid oat forms is a region in Mongolia and northwestern China.

## DNA markers and genomic technologies in evolution studies of Avena species

Studies of a complex of morphological features do not yield a complete picture of the evolutionary and systematic status of some species and forms of oats. The methods and approaches that use DNA markers and genomic technologies and are currently undergoing active development are promising for the study of polymorphism and phylogeny of oats.

Recently, there has been a number of works dealing with the molecular aspects of the evolution and phylogeny of the genus Avena (Fu, 2018; Peng et al., 2018, 2022; Latta et al., 2019; Ahmad et al., 2020; Liu et al., 2020; Fominaya et al., 2021; Jiang et al., 2021; Yan et al., 2021). These studies use various markers, such as the ITS1-5.8S rRNA-ITS2 sequences (Rodionov et al., 2005; Nikoloudakis et al., 2008; Nikoloudakis, Katsiotis, 2008; Tyupa et al., 2009), and external transcribed spacers (Rodrigues et al., 2017). These works have clarified a number of relationships between Avena species with different genomes (see the Table).

**Table 1.**
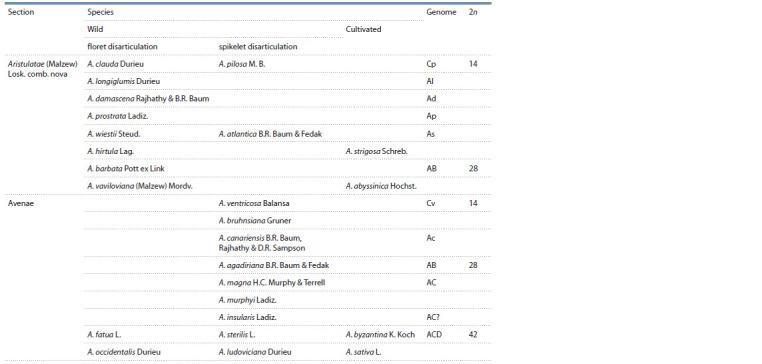
Speciation in the genus Avena L. (Loskutov, 2007)

When studying the relationships of hexaploid A. sativa, A. sterilis and diploid A. strigosa, retrotransposons and ITS sequences were used. An analysis of the ITS sequences showed very high homology in all three species, but FISH (fluorescent in situ hybridization) revealed differences in the position of nucleolar organizers (containing rDNA). According to the pattern of retrotransposon polymorphism, the hexaploid A. sativa turned out to be closer to A. sterilis than to the diploid A. strigosa (Tomas et al., 2016). Diploid wild species with their greatest diversity of forms in the western Mediterranean, presumably gave rise to the cultivated species A. strigosa, which is most widely distributed in the Iberian Peninsula. The wild species A. hirtula and A. wiestii most likely gave rise to the autotetraploid species A. barbata (Holden, 1979; Thomas, 1995).

Studies of the origin of polyploid oat species by the comparative analysis of the characteristics of the genome and DNA markers do not make it possible to draw unambiguous conclusions. For instance, C. Li et al. (2000) examined the occurrence of the species-specific satellite DNA ASS49 in 40 microsatellites and 4 minisatellites in diploids and polyploids in order to determine the species that was the diploid and tetraploid ancestor of the hexaploid oat. This comparison showed that the Ac genome of the diploid A. canariensis B.R. Baum, Rajhathy & D.R. Sampson is a more probable ancestral genome for A subgenomes of hexaploids rather than A. strigosa, which is usually regarded as such.

However, studies of other polymorphic markers give different results. For instance, the AFLP patterns of diploid, tetraploid, and hexaploid oat species show that it is not A. canariensis, but A. wiestii that is a more probable donor of A genomes for hexaploids with an ACD genomic constitution (Fu, Williams, 2008).

DNA samples were used to study the order of nucleotide sequences in species with different chromosome sets. The pAs102 probe obtained from A. strigosa (As) during in situ hybridization showed that sequences complementary to this probe are found in diploids with the A and C genomes, in tetraploids with the AC genome, and in hexaploids with the ACD genome. On the other hand, homologous sequences of the pAs102 probe were found in A. strigosa, A. longiglumis Durieu and A. sativa. A not very precise sequence is present in A. murphyi Ladiz. and is completely absent in other diploid species with the A and C genome variants (Linares et al., 1996, 1998).

It is assumed that the tetraploid wild species A. vaviloviana (A. abyssinica being its cultivated analog) is similar in some morphological features to the hexaploid wild species A. occidentalis Durieu originally found in Algeria. It has been established that, according to some morphological features, A. vaviloviana and A. abyssinica may be relics of the ancient African flora (Baum, 1971). In addition, B. Baum (1972) notes that morphological similarity was found between three species, namely A. vaviloviana, A. occidentalis and questionable from our point of view species A. septentrionalis, which A.I. Malzew (1930) attributed to the subspecies A. fatua growing in Siberia. These species are currently distributed on the territory in the form of broken (disjunctive) relict distribution ranges, and thus confirm the point of view, according to which the species of the genus Avena occupied entire (rather than fragmented) and diverse distribution ranges in the recent geological past, in comparison with the ranges of modern species (Baum, 1971; Rajhathy, 1971).

The genetic unity and interfertility of A. barbata and A. vaviloviana with A. abyssinica was confirmed by a genetic study of hybrids. It is assumed that the weedy species A. barbata, brought to Ethiopia together with barley grain, gave rise to the cultivated species A. abyssinica, which infests barley crops to this day (Thomas, 1995). On the basis of the material for the study and analysis of interspecific crossings, chromosome structure, morphological, biochemical characters and geographical distribution of species, it was concluded that diploid species with the As genome (hirtula-wiestii) were the ancestors of the group of tetraploid species with the AB genome (barbatavaviloviana- abyssinica), or AAʹ. In turn, the last group, evolutionarily unrelated to any other group of oat species, is a lateral branch of the genus Avena (Rajhathy, Thomas, 1974).

According to F.A. Coffman (1977), the ancestor of the diversity of cultivated hexaploid forms is A. sterilis, originating from the Asian continent. Apparently, the cultivated species A. byzantina originated from this species, and then A. fatua, a malicious weed that infests cultivated crops, appeared. The further consideration of the hexaploid species evolution showed that when studying translocations in oat chromosomes and the ratio of the geographical distribution of different forms using data cluster analysis, a high degree of genetic relationship was noted between A. byzantina accessions and forms of A. sterilis from northern Mesopotamia, on the one hand, and A. sativa accessions and forms of A. sterilis from eastern Anatolia, on the other hand (Zhou et al., 1999).

Further studies of all hexaploid species showed that translocations (97 %) were characteristic of A. sativa, in contrast to A. byzantina (11 %). As a result, it was suggested that two cultivated species, A. sativa and A. byzantina, were independently introduced into cultivation. A study of A. fatua and A. occidentalis showed that most forms of these species have the same translocations as A. sativa and, therefore, are regarded as side branches of oat evolution (Jellen, Beard, 2000).

Differences in genome size between species with different ploidy levels were significant and depended on genomic duplication, while differences in genome size within a certain ploidy level were mainly due to different genomic composition. The flow cytometry method made it possible to diagnose individual species and, in some cases, to establish intergenomic relationships between them (Yan et al., 2016).

By using 12 primer pairs of microsatellite markers of the chloroplast genome, 70 accessions of 25 Avena species from the VIR collection were analyzed. From 2 to 9 alleles were identified, and the average value of genetic diversity (H) amounted to 0.479. The differences in the length of alleles allowed the identification of 45 haplotypes The most polymorphic were the diploid species A. eriantha Durieu (A. pilosa M. B.) and A. ventricosa Balansa with the C genome, one of the diploid species with the As genome (A. atlantica B.R. Baum & Fedak) and tetraploid species A. insularis Ladiz. (AC genome) and A. agadiriana B.R. Baum & Fedak (AaBa genome). A. insularis, which is often regarded as the species closest to the hexaploid ones, is probably the most primitive among the tetraploid species with the AC genome, and cannot be the direct ancestor of the hexaploid species. This study identified new informative markers for the analysis of the chloroplast genome of the genus Avena and refined data on the phylogenetic relationships of oat species (Yan et al., 2016).

Based on the sequenced and annotated reference oat genome, quantitative trait loci (QTLs), economically valuable traits and those associated with grain quality in populations of cultivated A. sativa have been found and characterized. Strong and significant associations have been found between the positions of candidate genes and QTLs that affect heading date, as well as those that influence the concentrations of oil and β-glucan in the grain (Tinker et al., 2022).

In 2022, the genomes of three species of the genus Avena L. were completely sequenced; these were an allohexaploid cultivated oat species (Avena sativa, AACCDD, 2n = 6x = 42) and two of its close wild relatives: diploid A. longiglumis (AA, 2n = 14) and tetraploid A. insularis (CCDD, 2n = 4x = 28).

The publication of the results of the whole genome sequencing (Kamal et al., 2022) showed that the reference genome of cultivated oats A. sativa has a mosaic structure that differs sharply from the genomes of other members of the Poaceae family. This study showed that during the formation of a hexaploid oat species, at least 226 Mb of gene-rich regions from the C subgenome were translocated into subgenomes A and D, which is associated not with the loss of individual genes, but with a large number of translocations in the latter. In contrast to hexaploid common wheat, crosses between species with different ploidy and alien introgressions were extremely complex in the genus Avena, suggesting the presence of an incompatible genome architecture, which is an additional barrier preventing genetic improvement in A. sativa. Average expression values across transcriptome samples from six tissues showed that C subgenome genes were slightly less expressed (32.32 %) than those in the D (33.53 %) and A (33.76 %) subgenomes. A network approach revealed that genes from the C subgenome were found in divergent expression modules more frequently than their A and D subgenome homoeologues (Kamal et al., 2022).

Based on the sequenced and annotated reference oat genome, genome-wide recombination profiles were examined to confirm the presence of a large unbalanced translocation from chromosome 1C to chromosome 1A and a possible inversion on chromosome 7D, which are typical for oats (Tinker et al., 2022).

Subsequently, the time of divergence of three oat subgenomes was calculated. The divergence time of the A subgenome was ~47.3 thousand years ago, the C subgenome, ~47.0 thousand years ago, and the D subgenome, ~53.3 thousand years ago (Nan et al., 2023).

## A comprehensive evaluation of the diversity of species of the genus Avena using next generation sequencing (NGS) methods

A comprehensive study of a representative set of accessions from the collection of the VIR, demonstrating a wide ecological and geographical diversity of all four cultivated and 21 wild species of the genus Avena L. with different ploidy levels, showed that the diploid species A. bruhnsiana Gruner has a hybrid origin, i. e. is a notospecies, one of the ancestors of which was A. ventricosa, and the second, apparently, A. clauda. The karyotype of A. bruhnsiana is diploid (2n = 14) (Loskutov, Abramova, 2006), therefore it can be assumed that this is a homoploid hybrid. Judging by the diversity of rDNA sequences, A. clauda itself is probably also a homoploid hybrid: one of its main ribotypes is identical to that of A. pilosa, while the other is isolated (Gnutikov et al., 2022b).

Therefore, out of the four studied diploid oat species with the C genome, two are homoploid hybrids. It has also been found that species with two nucleolar organizers in the genome on different chromosomes often have at least two ribotypes, while A. ventricosa, which has one nucleolar organizer (NOR), has only one ribotype. This may indicate that rDNA homogenization proceeds within one NOR more effectively than that at loci located on different chromosomes. Perhaps this is due to the fact that one of the mechanisms of rDNA homogenization is associated with the conjugation of homologous chromosomes and, therefore, proceeds more effectively within one NOR than between NORs located on different chromosomes (Eickbush T.H., Eickbush D.G., 2007; Sochorová et al., 2018).

Among the studied C genome species of oats, there is one autotetraploid (2n = 28), perennial, cross-pollinated, narrowly endemic species from Algeria, A. macrostachya Balansa & Durieu. This species is considered the most ancient species of the genus Avena (Nikoloudakis, Katsiotis, 2008; Peng et al., 2008, 2010). Morphologically, this perennial species is a primitive representative of the genus Avena (Malzew, 1930). Some researchers even assigned it to the genus Helictotrichon Besser (Holub, 1958). Avena macrostachya differs from diploid oat species with the C genome by a symmetrical karyotype with the predominance of equal-armed chromosomes, an absence of diffuse heterochromatin, a predominantly pericentromeric location of C-positive bands, as well as the size and morphology of satellite chromosomes (Badaeva et al., 2010). As it turned out, a symmetrical karyotype is not characteristic of diploid species with the C genome. At the same time, large blocks of C-heterochromatin in the pericentromeric regions of chromosomes of this species indicate its relationship with the C genome species. This confirms that the C genome of A. macrostachya is of a special type, hence its designation CmCm (Rodionov et al., 2005). It was also believed that A. macrostachya could have a previously undescribed EE genome (Loskutov, 2007). Our analysis of NGS data for 18S-ITS1-5.8S rDNA sequences showed that A. macrostachya ribotypes are comparatively far from other existing C genome oats.

An analysis of the intragenomic rDNA polymorphism of diploid oat species with different variants of the A genome showed significant differences in the number of ribotypes, haplotypes, nucleotide diversity indices, genetic distance, and genetic differentiation (Rodionov et al., 2005).

The evaluation employed accessions with a high ecological and geographical diversity, which represented all variants of the A genome, i. e. the As (A. atlantica, A. hirtula, A. wiestii), Ac genome (A. canariensis), Ad (A. damascena Rajhathy & B.R. Baum), Al (A. longiglumis), and Ar (A. prostrata Ladiz.). Also, one species with the C genome, A. clauda, was taken into analysis. Sequences of 169 accessions revealed 156 haplotypes, of which seven haplotypes are common for two to five species. Sixteen ribotypes were identified, which consisted of a unique sequence with a characteristic set of single nucleotide polymorphisms and deletions. The number of ribotypes per species varied from one in A. longiglumis to four in A. wiestii. Although most of the ribotypes were species-specific, two ribotypes were found to be common for three species (one for A. damascena, A. hirtula, and A. wiestii, and the other one for A. longiglumis, A. atlantica, and A. wiestii), while a third ribotype was common for A. atlantica and A. wiestii. A characteristic feature of the ribotype of A. clauda, a species with the diploid C genome, is that two different ribotype families were found in this species. Some of these ribotypes are characteristic of species with the Cp genome, while others are closely related to ribotypes of the As genome. It means that A. clauda may be a hybrid of oats with the As and C genomes.

Despite the fact that the studied species of the genus Avena were diploids, it turned out that most of them contained several different rDNA families. A comparative study of rDNA patterns in individual species showed that an rDNA pattern, as a rule, is mosaic and species-specific in all cases. At the same time, oat species with the A genome can reflect hybridization events that took place in their evolutionary past as a way of their speciation (Loskutov et al., 2021; Gnutikov et al., 2022a).

A large set of landraces with unique, so-called segetal botanical varieties of cultivated oats was subjected to study. These forms are specialized weeds of emmer wheat and barley, which spread together with the grain of cultivated plants and weed crops. All these botanical varieties form a separate clade with a good level of support, while their differences are small (p-distance from 0.003 to 0.02). All of them are hexaploids with the ACD genome (Loskutov, Rines, 2011), however, it should be remembered that Sanger sequencing reveals only the most massive subgenome variant in the polyploid genomic set.

The NGS results revealed two ribotype families most represented in terms of the number of sequences in the polyploid genomic set, which are common for almost all studied botanical varieties related to A. fatua and A. sativa. These two ribotype families correspond to the sequences of the A genome and the D (A′) genome, which was previously assumed to be a variant of the A genome (Loskutov, 2007). At the same time, most of the ribotypes in these genomes were common for all the studied accessions

It is also of great interest that the C genome sequences were not found in the general pool of hexaploid sequences; they were located separately as a very small fraction, probably strongly altered by the processes of post-hybridization transformation. Similar data are confirmed by cytogenetic studies. The FISH method showed that the C subgenomes of polyploid oat species have lost most of the rDNA, and only very weak 35S rDNA-positive signals can be detected on them (Badaeva et al., 2010).

Using sequence-tagged DNA sequencing on the Roche 454 platform, intragenomic polymorphism of one of the 35S rRNA regions (18S rDNA fragment–ITS1–fragment of 5.8S rDNA) in three hexaploid Avena species with karyotypes AACCDD and the tetraploid species A. insularis (AAСС or CCDD) has been studied (Rodionov et al., 2020). Instead of the expected 50 % of C-variant ITS1 in A. insularis and 33 % of C-variant ITS1 in hexaploids A. fatua, A. ludoviciana, and A. sterilis, the actual rate of C subgenome specific ITSs comprised around 3.3 % of rDNA in A. insularis and 1.4–2.4 % of rDNA in hexaploid genomes. The 18S rDNA, ITS1 and 5.8S rDNA of the C subgenome origin were 10 times more variable than the same sequences from the A genome. Some of the C subgenome sequences contained deletions, including deletions in the 18S rRNA coding region

The results of FISH hybridization with pTa71 and pTa794 confirm the fact that polyploids lost a significant part of the 35S rDNA and 5S rDNA obtained from a diploid ancestor with the CC karyotype. The sequences of the ITS1 C subgenomes of polyploid species are diverse, but among them it is possible to single out the main (core) variant approximately equidistant from the ITS diploids carrying the Cv and Cp genomes. The results show that the loss of 35S rDNA C subgenomes occurs against the background of the accumulation of many single nucleotide substitutions (SNPs) and deletions accumulation in these sequences. In the “repressed” 35S rRNA loci of C subgenomes, multiple mutations were apparently not accompanied by homogenization of rDNA. Hence, there is a reason to believe that the processes of rDNA isogenization and the process of transcription/silencing are related phenomena (Rodionov et al., 2020).

Obtaining results using the method of locus-specific next generation sequencing (NGS) on the Illumina platform allowed for a phylogenetic analysis of representative intraspecific diversity of cultivated and wild species of the genus Avena. It has been established that diploid species with the A genome (variants of Al, Ap and As genomes) are in fact not primary diploids, but a kind of Mediterranean introgressive hybridization complex of species that sporadically enter into interspecific hybridization. It has been determined that the contribution of A. canariensis (considered to be a donor of the A genome for hexaploids) to the genomic constitution of hexaploids (ACD) is insignificant, and according to our data, it is of hybrid origin, as two ancestral species with close, but not identical ribotypes, took part in its formation. It has been established that the tetraploid cultivated species A. abyssinica most likely originated from the wild A. vaviloviana. At the same time, A. agadiriana, previously considered as an ancestor to A. abyssinica and its group of relatives, forms separate unique subgenomes (ribotype families). An analysis of the ways of domestication of three oat species A. abyssinica, A. sativa, and A. byzantina showed that the most widespread ribotype of the A. sativa hexaploid was inherited from A. ludoviciana, and the second most widespread, from A. magna H.C. Murphy & Terrell. The cultivated species A. byzantina has two unique ribotype families, most likely inherited from an extinct oat species or cryptospecies, which has not been discovered until now (Gnutikov et al., 2021, 2022c).

On a representative set of oat species (Avena L.), the origin of wild polyploid species was analyzed. The 18S– ITS1–5.8S rDNA region was used for NGS analysis. In polyploid oats, 15 major ribotypes were found (more than 1000 reads per rDNA pool). Pools of marker sequences of polyploid oat species were compared with sequences of putative diploid ancestors: A. atlantica (As genome), A. hirtula (As), A. canariensis (Ac), A. ventricosa (Cv), and A. clauda (paleopolyploid with the Cp genome and rDNA sequences related to the A genome). The results confirmed some earlier hypotheses about the origin of the polyploid species of the genus Avena. Tetraploid oats, which were previously identified as species with the AC genome, do have this genomic constitution. The data obtained do not support the hypothesis of CD genome recruitment in the tetraploid species A. magna, A. murphyi, and A. insularis. At the same time, D genome sequences were not found in tetraploid oats with the AC genome related to oats with the ACD genome

The sequences associated with the A genome may have been inherited from the As genomic species A. atlantica, while the sequences associated with the D genome were already formed in the hexaploid oat or were taken from an unknown ancestor related to A. clauda. It was found that AB tetraploid oats may have inherited their A genome ribotypes from A. atlantica (As1 ribotype), whereas their B genome ribotype is specific and may be derived from the A genome family. The A genome sequences in the ACD genome species of the genus Avena were probably inherited from A. murphyi (AC). The sequences associated with the C genome could be derived from the diploid species A. ventricosa. All hexaploid species show a different ribotype pattern from tetraploids; the main ribotypes of A. fatua, A. ludoviciana, and A. sterilis probably belong to the D group and are also common with one of the main ribotypes of the diploid species A. clauda (Gnutikov et al., 2023).

## Conclusion

Thus, on the basis of a comprehensive study of the complete intraspecific diversity of cultivated oat species from different zones of the distribution range and analysis of data on the geographical location of distribution ranges of forms and species, it was confirmed that the place of the greatest distribution and species morphogenesis in the genus Avena is located in the western part of the Mediterranean center of origin of cultivated plants, namely, on the territory of the northwestern part of the African continent and partly on the southwestern tip of Europe. It has been established that the process of hexaploid species formation also proceeded in the western part of the Mediterranean, and then, moving eastward, these forms began to occupy more and more areas in the South-West Asian center, forming a large intraspecific diversity of wild and weedy forms in transit to cultivated hexaploid oat species. Based on the analysis of the intraspecific diversity of landraces, the centers of morphogenesis of all cultivated oat species were specified.

The phylogenetic analysis of the representative intraspecific diversity of cultivated and wild Avena species, carried out using NGS methods, showed that diploid species with A genome variants are in fact not primary diploids, but a peculiar Mediterranean introgressive hybridization complex of species that sporadically enter into interspecific hybridization. It was established that the tetraploid cultivated species A. abyssinica had most likely originated from the wild A. vaviloviana. An analysis of the ways of A. sativa and A. byzantina domestication showed that the most widespread ribotype of the A. sativa hexaploid was inherited from A. ludoviciana, and the second most widespread one – from A. magna, while A. byzantina has two unique ribotype families, most likely inherited from an extinct oat species or a still undiscovered cryptospecies. Hexaploid wild species show a different pattern of ribotypes than tetraploids; the main ribotypes of A. fatua, A. ludoviciana, and A. sterilis probably belong to the D group and are also common with one of the main ribotypes of the diploid species A. clauda.

## Conflict of interest

The authors declare no conflict of interest.
